# Antibiotics Drive Expansion of Rare Pathogens in a Chronic Infection Microbiome Model

**DOI:** 10.1128/msphere.00318-22

**Published:** 2022-08-16

**Authors:** John J. Varga, Conan Y. Zhao, Jacob D. Davis, Yiqi Hao, Jennifer M. Farrell, James R. Gurney, Eberhard Voit, Sam P. Brown

**Affiliations:** a School of Biological Sciences, Georgia Institute of Technology, Atlanta, Georgia, USA; b Center for Microbial Dynamics and Infection, Georgia Institute of Technology, Atlanta, Georgia, USA; c Emory + Children’s Center for Cystic Fibrosis and Airway Disease Research, Atlanta, Georgia, USA; d Department of Biomedical Engineering, Georgia Institute of Technology and Emory University, Atlanta, Georgia, USA; University of Michigan-Ann Arbor

**Keywords:** antibiotic resistance, chronic infection, cystic fibrosis, experimental microbiome, infection microbiome

## Abstract

Chronic (long-lasting) infections are globally a major and rising cause of morbidity and mortality. Unlike typical acute infections, chronic infections are ecologically diverse, characterized by the presence of a polymicrobial mix of opportunistic pathogens and human-associated commensals. To address the challenge of chronic infection microbiomes, we focus on a particularly well-characterized disease, cystic fibrosis (CF), where polymicrobial lung infections persist for decades despite frequent exposure to antibiotics. Epidemiological analyses point to conflicting results on the benefits of antibiotic treatment yet are confounded by the dependency of antibiotic exposures on prior pathogen presence, limiting their ability to draw causal inferences on the relationships between antibiotic exposure and pathogen dynamics. To address this limitation, we develop a synthetic infection microbiome model representing CF metacommunity diversity and benchmark on clinical data. We show that in the absence of antibiotics, replicate microbiome structures in a synthetic sputum medium are highly repeatable and dominated by oral commensals. In contrast, challenge with physiologically relevant antibiotic doses leads to substantial community perturbation characterized by multiple alternate pathogen-dominant states and enrichment of drug-resistant species. These results provide evidence that antibiotics can drive the expansion (via competitive release) of previously rare opportunistic pathogens and offer a path toward microbiome-informed conditional treatment strategies.

**IMPORTANCE** We develop and clinically benchmark an experimental model of the cystic fibrosis (CF) lung infection microbiome to investigate the impacts of antibiotic exposures on chronic, polymicrobial infections. We show that a single experimental model defined by metacommunity data can partially recapitulate the diversity of individual microbiome states observed across a population of people with CF. In the absence of antibiotics, we see highly repeatable community structures, dominated by oral microbes. Under clinically relevant antibiotic exposures, we see diverse and frequently pathogen-dominated communities, and a nonevolutionary enrichment of antimicrobial resistance on the community scale, mediated by competitive release. The results highlight the potential importance of nonevolutionary (community-ecological) processes in driving the growing global crisis of increasing antibiotic resistance.

## INTRODUCTION

Physicians face two growing crises that impact their ability to treat bacterial infections. The first is widely recognized—the evolution of antibiotic resistance (1). The second receives less attention—chronic (long-lasting) infections that are more difficult to control (2–4). Chronic infections are globally a rising burden on health care systems due to increases in populations at risk (e.g., the elderly, people with diabetes or other diseases that can promote chronic wounds) (5). At-risk populations have deficits in host-barrier defenses and/or immune function that provide an opening for the establishment of infections, and these chronic infections are further complicated by changes in pathogen growth mode (e.g., formation of multicellular biofilm-like aggregates [6–8]) and development of complex multispecies communities (9).

To address the global challenge of chronic infections, we focus on a particularly well-characterized disease, cystic fibrosis (CF), where bacterial infections can persist for decades. CF is caused by mutations in the cystic fibrosis transmembrane conductance regulator (CFTR), an ion channel that conducts chloride and thiocyanate ions across epithelial cell membranes, leading to defective mucociliary clearance and polymicrobial infection (10, 11), resulting in eventual pulmonary failure (12, 13).

Traditionally, CF research and patient care have focused on a small cohort of opportunistic pathogens, highlighting a distinct successional pattern (14) characterized by peak prevalence of Haemophilus influenzae in childhood, Staphylococcus aureus during adolescence, and Pseudomonas aeruginosa in adulthood. In addition to the core pathogen species, 16S rRNA-encoding gene amplicon sequencing of expectorated sputum samples has revealed much more diverse communities including numerous bacteria that are commonly considered nonpathogenic in CF and that are normally associated with oral and upper-respiratory environments (15–19). The functional role of these nonpathogenic taxa in CF lungs is currently disputed (20). Epidemiological analyses have identified potentially positive roles, as higher lung function correlates with higher relative abundance of oral bacteria in sputum samples from both cross-sectional (21–23) and longitudinal studies (24). In contrast, *in vitro* experimental studies have suggested health risks of specific oral bacteria in the lung, due to the potential facilitation of pathogen growth (25, 26). A third interpretation is that oral bacteria found in sputum are simply the result of sample contamination with oral microbes during expectoration (27, 28). A number of approaches to address the sputum contamination issue have been taken, including mouth cleaning and sputum rinsing (29), as well as more invasive sampling techniques (subject to clinical need [28, 30–32]). Most recently, computational analysis of paired sputum and saliva samples from adults with established CF lung disease has demonstrated that saliva contamination during sample collection has a minimal quantitative impact on the community profile (33).

As a result of long-term bacterial infection, people with CF are exposed to high levels of antibiotics (34), both as maintenance therapy (35) and as treatment for exacerbations. In the context of a critical health challenge (an acute pulmonary exacerbation), health outcomes are variable; lung function can rapidly increase back to baseline values or remain at a new, lower baseline following antibiotic intervention. Unfortunately, a recent systematic review of 25 articles indicated little correlation between these variable clinical outcomes and antibiotic susceptibility test results for the target pathogen (36). Several factors for this disconnect have been proposed, including differences in bacterial physiology (37), nonrepresentative infection sampling (38, 39) and polymicrobial interactions (40). In a microbiome context, epidemiological studies indicate variable outcomes of antibiotic treatment, ranging from minimal impact on microbiome structure (15, 41, 42) to target pathogen declines, microbiome structural changes (43–46), and risk of subsequent infection (47). However, there is a fundamental confounding factor in these epidemiological studies, as antibiotic exposures are themselves dependent on the microbiome state of the patient. Specifically, the detection of pathogens within the microbiome will dictate antibiotic choice (48).

Here, we seek to overcome this confounding impact of pathogen detection through the development and clinical benchmarking of an experimental infection microbiome model. Using this model, we seek to address a number of broad and overlapping questions concerning the determinants of infection microbiome structure: (i) Can a single experimental model generate multiple alternate infection microbiome states? (ii) What are the impacts of independent pathogen and antibiotic manipulations on microbiome structure? (iii) Can antibiotics drive pathogen expansion and community diversification, via competitive release? (iv) Do antibiotics promote facilitatory species interactions?

**FIG 1:**
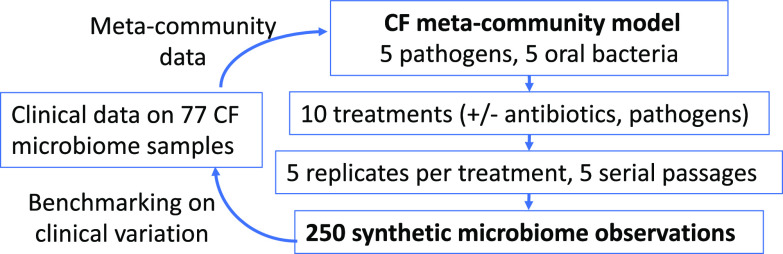
Schematic outline of the CF meta-community approach. All experiments are derived from a 10-species menu that captures the majority of CF microbiome diversity across a cohort of 77 people with CF (57) and is consistent with microbiome content across the CF literature (15–19, 21–24). The 10-species meta-community is exposed to 10 treatments (in 5× replication) and propagated for 5 serial passages. The experimental design results in 250 individual synthetic microbiome observations.

While most experimental polymicrobial models of CF have focused on two-species pathogen interactions (49–51), some studies have developed up to six-species models (52, 53). These more complex models have demonstrated that species antibiotic susceptibility is not impacted by community context (53), but their use of rich media (to facilitate single-species comparisons) raises the issue of relevance to the *in vivo* context of growth in sputum (54). Our experimental approach begins with a “synthetic sputum” that recreates the biochemical and physical conditions of the sputum found in CF lungs (55, 56). We then add defined combinations of the 10 most abundant bacterial species on the meta-community scale: 10 species that together account for over 85% of the observed bacterial diversity within the CF lung in a 77-person cohort (57). Five of these species are established human pathogens (S. aureus, P. aeruginosa, H. influenzae, Burkholderia cenocepacia, and Achromobacter xylosoxidans), while the rest are oral microbes frequently found in CF lungs. To underline that our 10-bacterial species model captures observed CF diversity at the meta-community (multipatient) scale, we refer to this model as the CF meta-community model (see schematic in Fig. 1) and view this model as representing the primary menu of organisms from which individual patient microbiomes form. We hypothesize that this single experimental model can self-organize into multiple alternate community states that approach the diversity of microbiome states observed across individuals with CF. The existence of alternate community states has recently been quantified by epidemiological analyses identifying five (58) or eight (59) distinct “pulmotypes” across cohorts of people with CF.

Replicate communities are cultured anaerobically to capture oxygen-depleted conditions within mucus plugs (60–62). We show that under our *in vitro* model infection conditions, oral bacteria form stable communities that suppress the growth of multiple pathogen species, and this competitive suppression is reduced by controlled antibiotic exposures, leading to multiple alternate pathogen-dominant outcomes, the emergence of facilitatory species interactions, and the nonevolutionary enrichment of antibiotic resistance.

## RESULTS

### In the absence of antibiotics, commensal anaerobes dominate over CF pathogens.

**FIG 2:**
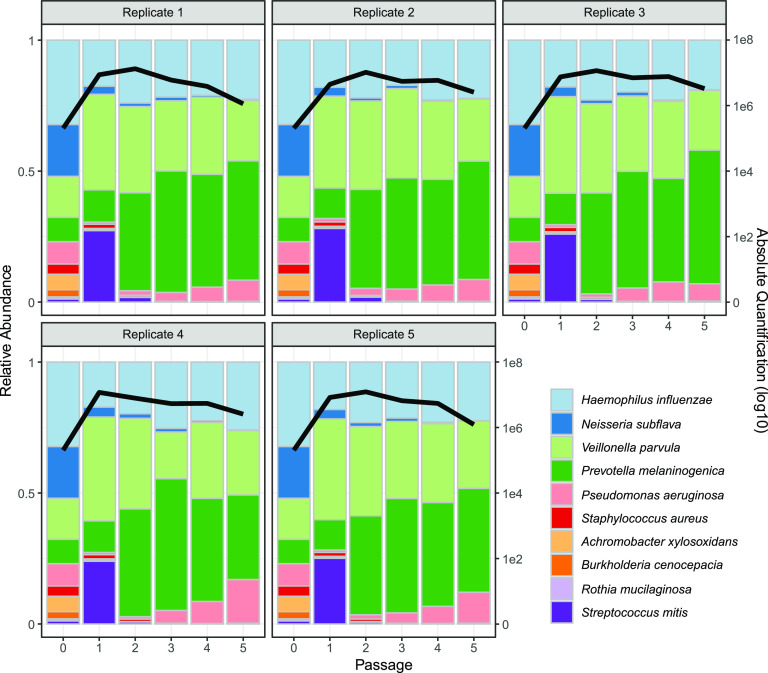
The 5-fold replicated synthetic CF microbiomes converge toward a single stable state in the absence of antibiotic perturbations. Five replicate synthetic microbiomes were grown anaerobically in artificial sputum medium. The community composition was estimated by 16S rRNA gene amplicon sequencing at time 0 and at every 2-day passage (*x* axes) into fresh medium (10% transfer of 2 mL culture volume). The colored bars represent the relative abundance of each species in the community (left *y* axis), while the black line represents the total bacterial abundance per mL (right *y* axis, log scale). Each panel represents a separate replicate experiment. Strain information is provided in Table 1 (our default P. aeruginosa strain is mucoid PDO300).

Experiments performed in the absence of antibiotics demonstrated a consistent and reproducible community structure, characterized by population expansion during the initial 48 h and a composition primarily consisting of Prevotella melaninogenica, H. influenzae, and Veillonella parvula (Fig. 2). At 48 h, the total bacterial density averaged about ~7.7 × 10^6^ CFU/mL (± 2.0 × 10^6^ standard deviation [SD]), which falls within the broad range of reported bacterial densities in sputum in clinical studies (typically between 10^4^ and 10^9^ CFU/mL [57, 63, 64]). From passage 3 onward, each replicate showed a high degree of stability through time, in terms of both total abundance and relative composition. Across replicates, we also see a striking convergence in microbiome structure. To assess consistency across the 5 replicates, we calculated coefficients of variation (CV = standard deviation/mean) for each species’ total abundance, all showing underdispersion (i.e., standard deviation less than the mean, with an average species CV of 0.46 at the end of the experiment; see Fig. S1 in the supplemental material), consistent with stabilizing ecological forces limiting variation in species densities across replicates.

10.1128/msphere.00318-22.5 FIG S1 Coefficient of variation (CV) in species abundances across replicates. CVs under different treatments through time. Stacked bars represent the CV of each individual species. CV is calculated as the standard deviation (across replicates) at each passage divided by its mean. Download FIG S1, EPS file, 0.3 MB. Copyright © 2022 Varga et al. 2022 Varga et al. https://creativecommons.org/licenses/by/4.0/ This content is distributed under the terms of the Creative Commons Attribution 4.0 International license.

**FIG 3:**
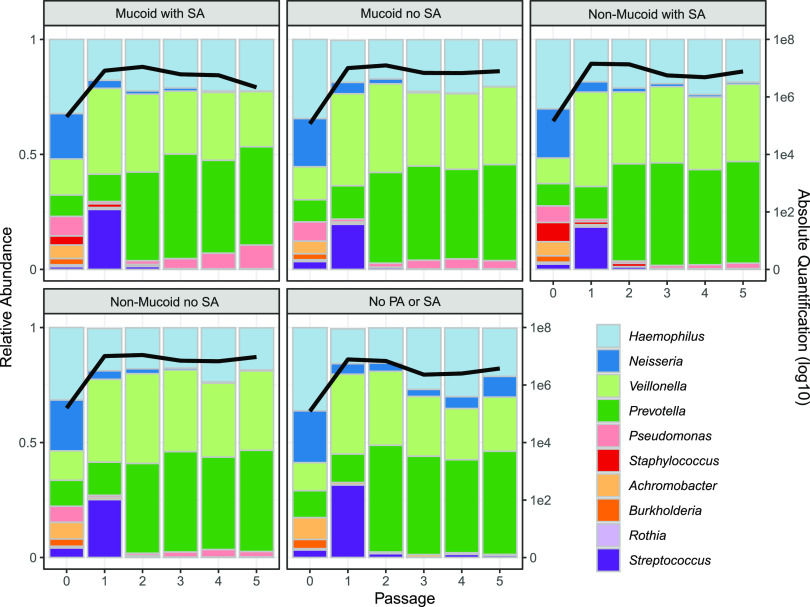
Varying the pathogen composition has minimal impact on community composition. Each panel represents the average of 5 replicates in the absence of antibiotics; the mucoid PA with SA panel is the average from Fig. 1. Figure details are the same as those described for Fig. 1. Data on individual replicates per treatment are presented in Fig. S2. Mucoid and nonmucoid PA, P. aeruginosa strains PAO1 and PDO300, respectively. SA, S. aureus.

The results in Fig. 2 point to a robust community structure in the absence of perturbations, consistent with the frequent dominance of oral bacteria in individuals with higher lung function but far from capturing the diversity of microbiome structures observed across the broader CF community; in particular, our results do not recapitulate the common observation of variably pathogen-dominated microbiomes (22, 24, 32, 57, 65). To assess the role of variable pathogen strain identity or presence/absence, we repeated the experiments in Fig. 2 with manipulations of the two most prevalent pathogens of people with CF, P. aeruginosa and S. aureus. Specifically, we manipulated the biofilm-forming ability of P. aeruginosa (mucoid PDO300 versus wild-type, nonmucoid PAO1 versus no P. aeruginosa) and also the presence/absence of S. aureus. In light of previous experimental work demonstrating that single-locus changes impacting biofilm phenotypes (such as mucoidy) can have dramatic community ecological impacts exceeding the impact of species removal (66), we hypothesized that the presence/absence of mucoidy would generate substantial community shifts, exceeding removal of S. aureus and/or P. aeruginosa. In contrast to this hypothesis, we found very small quantitative variations in community structure under all pathogen manipulations (Fig. 3) and no support for the prediction of a greater impact of mucoidy versus species removal (Table S1). Across all pathogen treatments, we observed overall the same qualitative pattern as in Fig. 2 with consistent dominance by H. influenzae, *P. melaninogenica*, and *V. parvula* (Fig. 3, Fig. S2).

10.1128/msphere.00318-22.1 TABLE S1 Differences in community structures across pathogen treatments (Fig. 3 data). The analysis of similarity (ANOSIM *R*) statistic captures the ratio of between-group to within-group variances; as *R* approaches 1, more variance is found between groups than within groups (114). Passage 0 (inoculum) is omitted from the analyses. All but one *R* value is significant via permutation tests at a *P < *0.05. Applying the more conservative convention of *R > *0.4 for significance (120, 121), we find no significant effects of any pathogen treatment. Download Table S1, DOCX file, 0.02 MB. Copyright © 2022 Varga et al. 2022 Varga et al. https://creativecommons.org/licenses/by/4.0/ This content is distributed under the terms of the Creative Commons Attribution 4.0 International license.

10.1128/msphere.00318-22.6 FIG S2 Compositional and total abundances across all treatments (columns) and replicates (rows). For details, see legend of Fig. 1. Download FIG S2, EPS file, 0.9 MB. Copyright © 2022 Varga et al. 2022 Varga et al. https://creativecommons.org/licenses/by/4.0/ This content is distributed under the terms of the Creative Commons Attribution 4.0 International license.

### Antibiotics skew community structure toward pathogen expansion and dominance.

**FIG 4:**
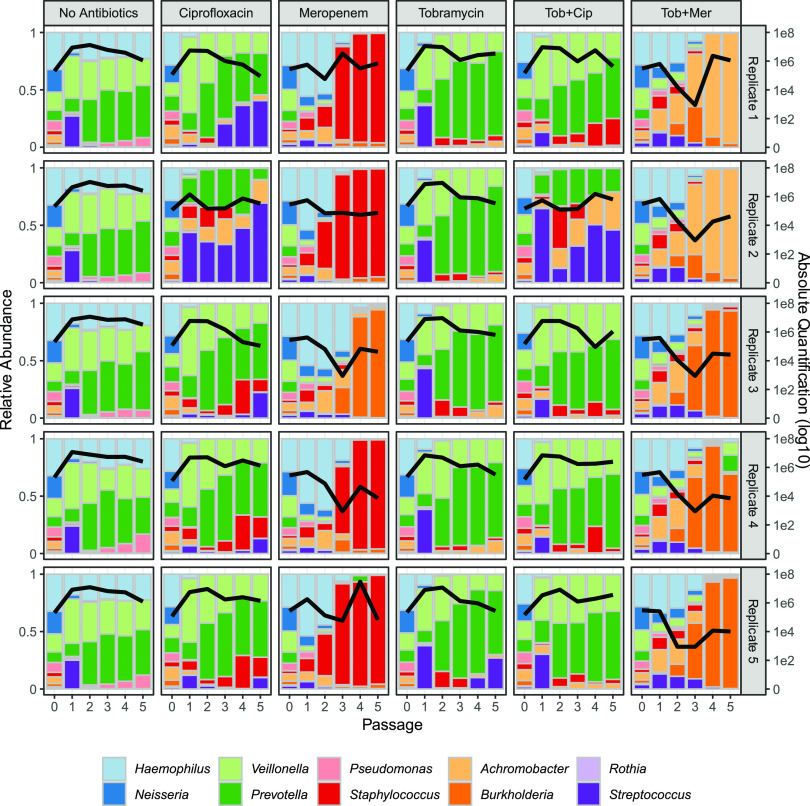
Antibiotic treatments produce large community fluctuations and alternative community states. Columns represent distinct antibiotic treatments (the first “no antibiotics” control column is reproduced from Fig. 1), and rows represent 5 replicates. The left axes measure community composition (bar charts); the right axes measure total bacterial abundance per mL (black lines). Experimental procedures, sampling, and analysis were performed as described for Fig. 1. Fresh antibiotics were resupplemented at each passage. Total abundance data by species are presented for each treatment and time point in Fig. S3.

Having established the repeatability and stability of the community in the absence of antibiotics, we then assessed the impact of antibiotic treatment on community structure. To test our hypothesis that antibiotic exposure will induce substantial community perturbations, communities were continually challenged with 3 individual antibiotics and 2 pairs commonly used in the CF clinic (tobramycin, meropenem, ciprofloxacin, tobramycin and meropenem, tobramycin and ciprofloxacin) (34, 67) using concentrations observed *in vivo* during therapeutic administration (68–72). Consistent with our hypothesis, antibiotic exposures resulted in dramatically different outcomes across treatments and replicates, compared to the antibiotic-free communities (Fig. 4, Fig. S1). To quantify community-scale impacts of antibiotic perturbations (compared to the no antibiotic control treatment, Fig. 2), we use the analysis of similarity (ANOSIM) *R* metric, revealing significant impacts on community structure, exceeding the impacts of pathogen treatments (Fig. S3). Antibiotic effect sizes range from modest impacts of tobramycin (mirroring clinical data [46, 73]) to substantial impacts for treatments involving meropenem.

10.1128/msphere.00318-22.7 FIG S3 Differences in community structures across experimental treatments and clinical data. The analysis of similarity (ANOSIM *R*) statistic captures the ratio of between-group to within-group variances; as *R* approaches 1, more variance is found between groups than within groups (114). Green boxes are comparisons between absolute abundances of the “no drug” reference treatment (PDO300 with SA, Fig. 1) and experimental manipulations (pathogen treatments, Fig. 3, and drug treatments, Fig. 4). The pink box is the comparison between relative abundances of all experimental conditions (Fig. 2 to 4) and all clinical conditions. Passage 0 (inoculum) is omitted from the analyses. All *R* values are significant with *P < *0.05 (permutation test). Applying the more conservative convention of *R > *0.4 for significance (120, 121), we find that only the antibiotic treatments achieve significant levels of community differentiation. Download FIG S3, EPS file, 0.2 MB. Copyright © 2022 Varga et al. 2022 Varga et al. https://creativecommons.org/licenses/by/4.0/ This content is distributed under the terms of the Creative Commons Attribution 4.0 International license.

The compositional presentation in Fig. 4 highlights that the same antibiotic treatment often leads to distinct pathogen dominance across replicates. For example, under meropenem 4 out of 5 replicates result in persistent S. aureus dominance, while one replicate shows persistent B. cenocepacia dominance. One possibility is that these distinct endpoints represent alternative stable states, implying stabilizing ecological forces sending separate replicates toward B. cenocepacia dominance (and S. aureus absence) or S. aureus dominance (and B. cenocepacia absence), dependent on fluctuations in initial conditions (74). To further investigate this claim, we turn to taxon absolute abundance data to test the “alternative stable states” prediction of a negative correlation between B. cenocepacia and S. aureus absolute abundances across replicates. Figure S4 presents absolute abundance data for each taxon, treatment, and replicate through time. Under the meropenem data (Fig. S4C) we can see substantial variation in the final abundance of B. cenocepacia and S. aureus (see also Fig. S1). However, across final abundances of these two taxa, there is no negative correlation between the absolute density of B. cenocepacia and S. aureus (Pearson’s correlation coefficient [correl coeff] = 0.026, *P* = 0.967). Under the meropenem/tobramycin treatment we see a similar pattern of variable dominance between Achromobacter xylosidans and B. cenocepacia (see Fig. 4, Fig. S1 and S4F), but again, no negative correlation across replicates in final absolute abundances (Pearson’s correl coeff = 0.310, *P* = 0.611). In light of this analysis, our data rule against alternative stable states. Given that our data do not reflect alternate stable states, one potential explanation for the pattern of the variable dominance across replicates under drug exposure is relaxed ecological regulation resulting in increased cross-replicate variation in species abundance.

10.1128/msphere.00318-22.8 FIG S4 Temporal absolute abundances for all treatments (panels A to F), taxa (subpanels) and replicates (colored lines). Data are plotted for each species under each antibiotic condition (A to F). The *x* axis represents passage, and the *y* axis represents absolute abundance per mL. Individual replicates are connected by individually colored lines. Download FIG S4, EPS file, 1.2 MB. Copyright © 2022 Varga et al. 2022 Varga et al. https://creativecommons.org/licenses/by/4.0/ This content is distributed under the terms of the Creative Commons Attribution 4.0 International license.

**FIG 5:**
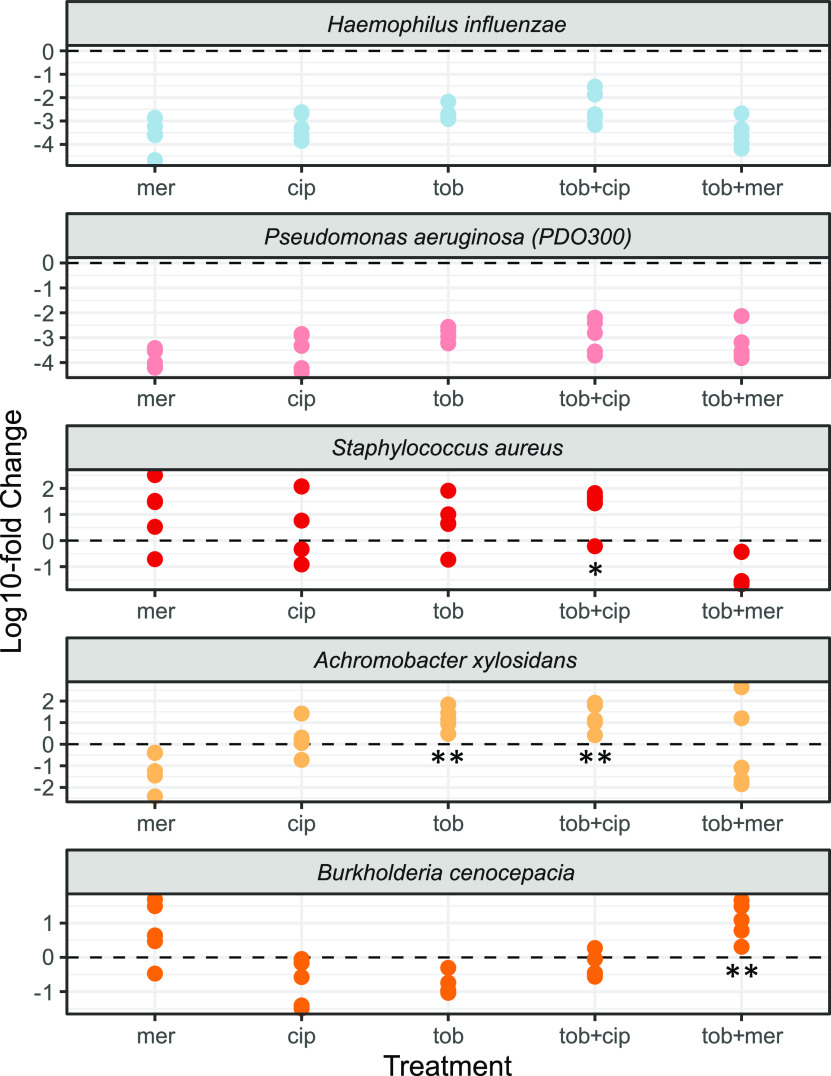
Absolute pathogen densities are variable and often increased under antibiotic exposures. Each dot corresponds to the fold change difference of an individual replicate of species-specific final time point absolute density under defined antibiotic treatments, compared to the mean value of the no-antibiotic control (data redrawn from Fig. 3). Mer, meropenem; cip, ciprofloxacin; tob, tobramycin. Asterisks denote significantly higher final densities in the presence of antibiotic compared to antibiotic-free controls (competitive release; one-tailed Wilcoxon test; *, *P < *0.05; **, *P < *0.01).

Figure 4 indicates large shifts in response to antibiotic treatments, but compositional analysis alone cannot separate the relative importance of differential survival versus differential expansion. Using absolute abundances (Fig. S4), we can now test whether pathogens undergo competitive release (expansion, following removal of competitors [75–77]) in response to antibiotic exposure, by assessing whether the final pathogen density is greater in the presence of antibiotic than in its absence (Fig. 5). Comparing densities in the presence/absence of antibiotics, we find evidence for significant and substantial (>100-fold in some replicates) antibiotic-dependent amplification via competitive release of S. aureus, B. cenocepacia, and A. xylosoxidans under specific antibiotic exposures (Fig. 5). In contrast, there is evidence of significant suppression of H. influenzae and P. aeruginosa in all antibiotic treatments (Fig. 5; two-tailed Wilcoxon test, *P < *0.01), together with Neisseria subflava in all treatments as well as *V. parvula* and *P. melaninogenica* in all meropenem treatments (Fig. S5).

10.1128/msphere.00318-22.9 FIG S5 Absolute microbe densities across antibiotic exposures. Each dot corresponds to an individual replicate of species-specific initial (inoculum) and final time-point absolute density under defined antibiotic treatments (data redrawn from Fig. 4). abx_free, no antibiotic condition; mer, meropenem; cip, ciprofloxacin; tob, tobramycin. Asterisks denote significantly higher/lower final densities in the presence of antibiotic compared to antibiotic-free controls (two-tailed Wilcoxon test; *, *P < *0.05; ** *P < *0.01). Download FIG S5, EPS file, 0.5 MB. Copyright © 2022 Varga et al. 2022 Varga et al. https://creativecommons.org/licenses/by/4.0/ This content is distributed under the terms of the Creative Commons Attribution 4.0 International license.

### Antibiotic susceptibility explains community composition on a functional scale but not on a taxon scale.

The simplest hypothesis to account for the substantial impacts of antibiotic exposures on community structure (Fig. 4 and 5, Fig. S3) is that antibiotics present a survival filter through which only resistant organisms can pass. Under this model, the community structure after antibiotic treatment is simply the product of whether or not each taxon can grow in the antibiotic(s) administered.

**FIG 6:**
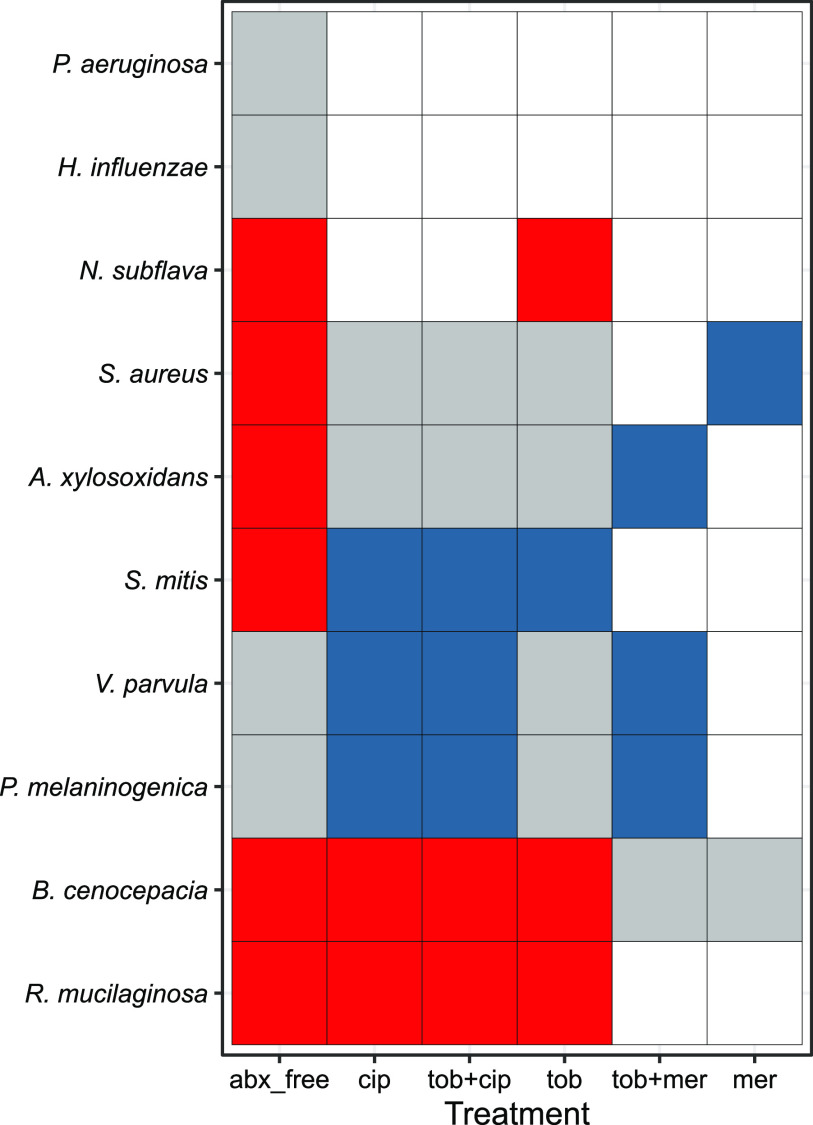
Antibiotic resistance testing does not consistently predict species presence/absence in a community context. For each species-drug combination, we assessed predicted survival (MIC in rich medium [Table S2] > experimental concentration) and observed survival (relative abundance of at least 1% averaged across all five replicates at the final time point [Fig. 4]). True-positive cases (predicted and observed present) are coded in gray; true negatives (predicted and observed absent) are in white. False positives (predicted present, observed absent; evidence for competition) are in red, and false negatives (predicted absent, observed present; evidence for facilitation) are in blue. Species order was determined through clustering via stringdist (118). In Discussion we address the caveat that single-species MIC measures are taken under distinct growth conditions.

To assess the survival filter hypothesis, we derived antibiotic susceptibility measures (MICs) under standard growth conditions that allowed the more fastidious strains to grow independently (Table S2) and used these data to predict experimental responses to defined antibiotic exposures (Fig. 6). Figure 6 illustrates that the drug-susceptible P. aeruginosa lab strain PDO300 behaves as predicted by the survival filter hypothesis: it is present in the absence of treatment but then absent (average relative abundance is <1%) in the presence of antibiotics. The same is true for H. influenzae.

10.1128/msphere.00318-22.2 TABLE S2 Antibiotic susceptibility in rich medium. MICs (in μg/mL) of synthetic community members were determined in rich medium. Download Table S2, DOCX file, 0.01 MB. Copyright © 2022 Varga et al. 2022 Varga et al. https://creativecommons.org/licenses/by/4.0/ This content is distributed under the terms of the Creative Commons Attribution 4.0 International license.

However, for multiple examples the ability to resist antibiotics (in a standard clinical assay [78, 79]) did not predict the presence/absence of the species after treatment. In red, Fig. 6 displays cases where the species was predicted to be present (given MIC resistance data, Table S2) but was nevertheless absent in the final community. This pattern is suggestive of an additional role for microbe-microbe competitive interactions in shaping community structure and was observed for 6 of the 10 taxa, and most often in the absence of antibiotics. Conversely, blue regions in Fig. 6 identify cases where the pathogen was predicted from MIC data to be unable to grow in the allocated antibiotic and yet was present in the multispecies community experiment in at least 1 community. This pattern is indicative of antibiotic-dependent faciliatory interactions, where other species aid the focal species to survive under antibiotic insult, for example, via antibiotic detoxification (80–85).

**FIG 7:**
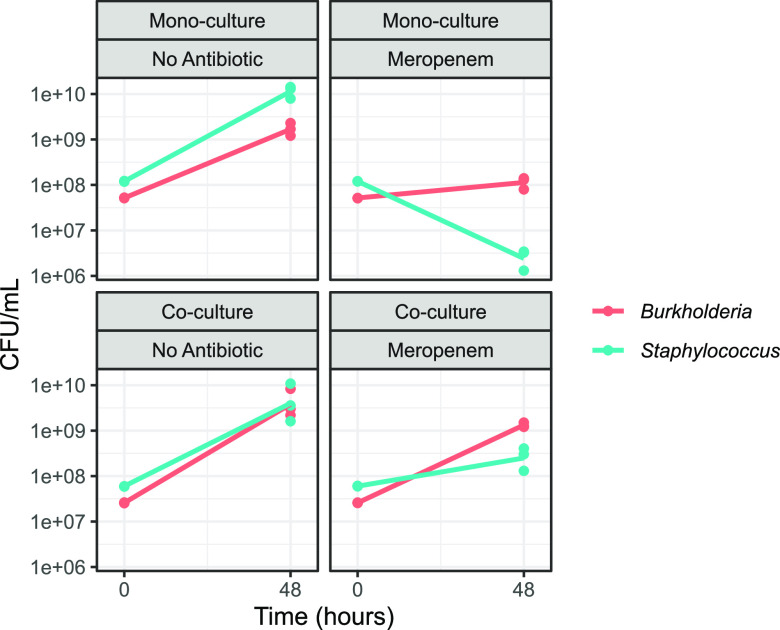
S. aureus growth in meropenem is facilitated by coculture with B. cenocepacia. Experiments were conducted in rich medium (Tryptone Soya Yeast Extract [TSYE] broth) in room air, in the presence or absence of 10 μg/mL meropenem and for each species either grown alone (monococulture) or together (coculture) in a 96-well plate with hourly shaking. At 0 and 48 h, cells were serially diluted and plated at concentrations of 10^−2^ to 10^−7^ onto either mannitol salt agar (for S. aureus) or LB agar with 500 mg/L gentamicin (for B. cenocepacia).

To assess the role of antibiotic-dependent facilitation, we focus on the meropenem treatment (far right column, Fig. 6), which indicates that the ability of S. aureus to grow in an otherwise lethal dose of meropenem is due to facilitation by B. cenocepacia. B. cenocepacia carries multiple β-lactamase enzymes (86) that are potentially capable of degrading meropenem and therefore enable S. aureus to grow in this environment. To test the facilitation hypothesis, we culture B. cenocepacia and S. aureus alone and in coculture (using rich media to allow monoculture comparisons) in both the presence and absence of meropenem (Fig. 7). In monoculture we find that S. aureus growth is limited by meropenem (paired one-tailed *t* test on S. aureus final density ± meropenem, *P = *0.003), consistent with MIC data (Table S2). In contrast, in coculture we find that S. aureus growth in meropenem is rescued by coculture with B. cenocepacia (paired one-tailed *t* test on S. aureus final density in meropenem, ± B. cenocepacia, *P = *0.020), consistent with antibiotic-dependent facilitation.

**FIG 8:**
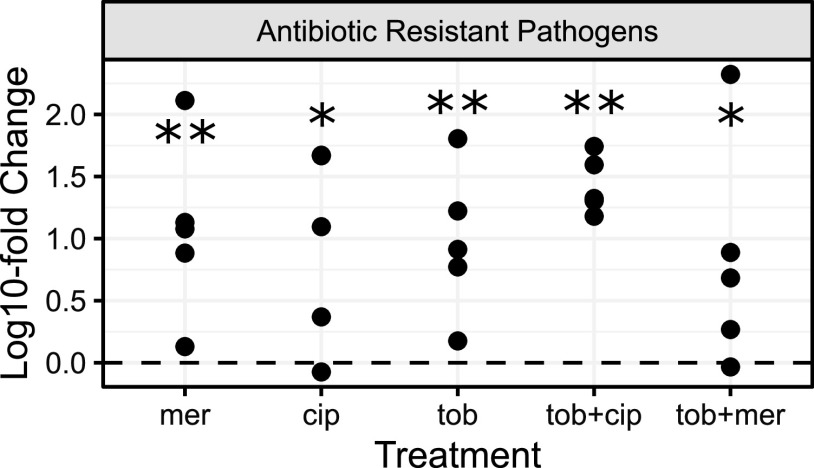
Drug-resistant pathogens are consistently enriched as a functional class across all drug treatments. Fold change differences for the sum of drug-resistant pathogens (B. cenocepacia, A. xylosoxidans, and *S aureus*) compared to no-antibiotic control; details as in Fig. 4. Asterisks mark significant competitive release; one-tailed Wilcoxon test; *, *P < *0.05; **, *P < *0.01.

In light of the inability of antibiotic resistance data to reliably predict community structure at the species scale (Fig. 6), we next asked whether the resistance data are predictive at a broader, functional scale. Pooling drug-resistant pathogens together (S. aureus, B. cenocepacia, and A. xylosoxidans), we find consistent enrichment (19- to 41-fold on average per treatment) across all drug exposures (Fig. 8), indicating a consistent enrichment of more problematic organisms following antibiotic exposure. In the discussion we explore potential contributing reasons other than community ecological interactions for the disconnect between MIC predictions on the species scale and observed community presences (Fig. 5).

### Community compositions across all antibiotic treatments are consistent with diversity across clinically observed *in vivo* communities.

**FIG 9:**
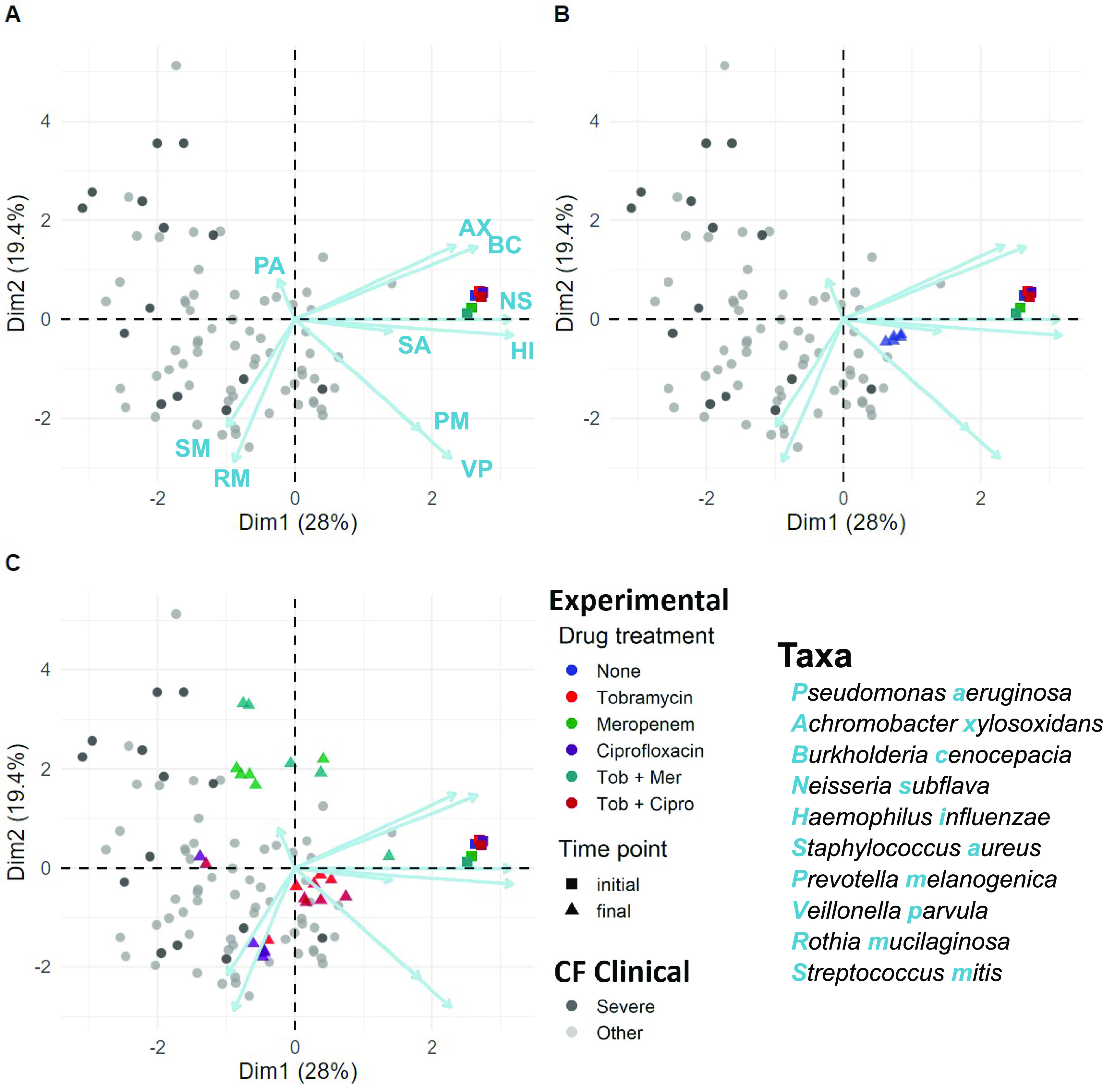
Antibiotics drive pathogen enrichment in experimental microbiomes, producing community structures that overlap clinical sputum communities. PCA visualization of experimental microbiome data (colored triangles and squares, summarizing data in Fig. 4) plus clinical microbiome data across a cohort of 77 people with CF (gray/black circles, black/severe signifies low lung function [57]). (A) Squares illustrate experimental initial conditions. (B and C) Triangles are final compositions after 5 serial passages (10 days), in the absence (B) or presence (C) of antibiotics. Colors denote experimental condition (see key). Each experimental treatment is replicated 5-fold, producing highly repeatable dynamics in the absence of antibiotics (blue triangles, B) and variable pathogen enriched outcomes following antibiotic treatment (C). Antibiotics were supplemented at each passage at clinically relevant concentrations (meropenem, 15 μg/mL; tobramycin, 5 μg/mL; ciprofloxacin, 2.5 μg/mL). Each point is a single microbiome sample (species resolution for clinical samples via the DADA2 plugin in QIIME2 [57, 119]). Ordination is the PCA of centered log-ratio transformed relative abundances.

We finally ask, how do our *in vitro* synthetic microbiomes compare with the diversity of microbiome structures observed in people with CF? We begin with a principal-coordinate analysis (PCA) ordination plot to visualize experimental data (initial and final time points from Fig. 4) alongside clinical data (Fig. 9).

Figure 9A illustrates that our initial 10-species inocula (colored squares) are not representative of individual patient microbiome states (gray/black circles), reflecting their derivation from the typical meta-community state of populations with CF (Fig. 1). Figure 9B highlights the repeatable endpoint microbiomes in the absence of antibiotics (blue triangles), approaching commonly observed oral microbe-dominated states (57). Figure 9C illustrates the more divergent states resulting from antibiotic perturbations. Contrasting clinical versus pooled experimental data, we see intermediate levels of community differentiation (ANOSIM *R *= 0.28), intermediate between the impacts of pathogen (Fig. 3) and antibiotic (Fig. 4) manipulations (Fig. S3).

**FIG 10:**
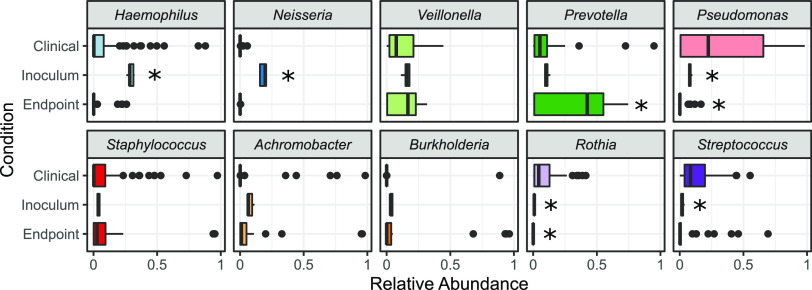
Most endpoint experimental taxa fall within the range of clinically observed relative frequencies. The relative abundances of taxa in synthetic microbiome inocula and endpoints (30 samples) compared to 77 clinical cohort observations (57). The box represents the interquartile range (from 25% to 75% of samples), with the horizonal line at the median. Outliers are represented as dots (two-tailed Welch’s *t* test versus clinical data with Bonferroni multiple testing correction; *, corrected *P < *0.001).

Building on the overview provided by Fig. 9, we now look at a more granular level and ask for each taxon whether species relative abundances in our experimental model fall within the range of clinical variation from our previous clinical study (57). In Fig. 10 we first assess our metacommunity inoculum condition (time zero in Fig. 2 and 4) against the yardstick of clinical variation and, unsurprisingly, see a substantial number of taxon misses (5 out 10 species abundance in the inocula is distinct from clinical data; Welch’s *t* test, *P < *0.001), reflecting that our metacommunity initial conditions are not well matched to the typical profiles of individual sputum samples (Fig. 1 and 9). We next assess experimental community states after 5 serial passages (final time points across all treatments) and find a better match with clinical data. Three of the five taxon misses move within clinical variation, while one taxon (*P. melaninogenica*) moves outside clinical variation, resulting in seven out of ten taxa where our experimental model produces ranges of relative abundances that do not significantly differ from benchmark clinical data (Fig. 10). We find that our model significantly overrepresents *Prevotella* and underrepresents Pseudomonas and *Rothia.* These misses provide an opportunity to improve our model in future work, by pointing toward an environmental mismatch on oxygenation (with the strict anaerobe *P. melaninogenica* benefitting and the facultative anaerobes P. aeruginosa and Rothia mucilaginosa suffering from the anaerobic atmosphere). This pattern of misses suggests that the distribution of oxygenation experienced clinically by CF microbiomes is more oxygenated than that provided by our anaerobic chambers with only brief exposures to oxygen every 48 h.

## DISCUSSION

Our results show that in the absence of antibiotic perturbations, our defined 10-species synthetic CF microbiome community follows a highly repeatable path to a stable community composition (Fig. 2, 3, and 9B). In contrast, antibiotic treatments resulted in substantial community shifts (Fig. 4), featuring both competitive release of previously rare pathogens (Fig. 5) and emergent facilitatory interactions (Fig. 7). Under antibiotic treatment we observed distinct trajectories across both drugs and replicates (Fig. S2 and S4), dispersed through a broad range of observed CF community structures, including alternate pathogen-dominant states (Fig. 9). Table S3 summarizes our results in light of motivating hypotheses.

10.1128/msphere.00318-22.3 TABLE S3 Summary of hypothesis tests conducted in this study. Hypotheses (italic text) are organized by topic area (underlined text). Hypotheses in grey are rejected under the specific experimental conditions outlined in our study and may still apply in other contexts. Previous literature citations identify work that is broadly relevant to the topic identified, and citations do not imply support for the specific hypotheses outlined. Download Table S3, DOCX file, 0.02 MB. Copyright © 2022 Varga et al. 2022 Varga et al. https://creativecommons.org/licenses/by/4.0/ This content is distributed under the terms of the Creative Commons Attribution 4.0 International license.

Our results highlight that standard antibiotic resistance MIC data (Table S2) often fail to predict individual species presence following antibiotic exposure (Fig. 6), with predictions showing both false positives and false negatives. A simple general explanation for departures from the ecological filter hypothesis is the presence of significant ecological interactions among species. Under this framework, false positives are evidence for suppressive interactions, suggesting that, for example, S. aureus fails to grow in the antibiotic-free environment because it is out-competed by one or more of the other taxa. Conversely, false negatives are evidence of facilitation, suggesting for instance, that the ability of S. aureus to grow in an otherwise lethal dose of meropenem is due to facilitation from another species in the community. In agreement with this hypothesis, we find that B. cenocepacia shows meropenem-dependent facilitation of S. aureus growth, of sufficient magnitude to rescue S. aureus growth in superinhibitory concentrations of antibiotic (Fig. 7).

While the combination of antibiotic resistance and species interactions is a candidate explanation for our results (Fig. 7), other factors are potentially at play. First, we again note the important caveat that the MIC estimates were derived using standard growth-promoting rich culture assays, which are known to generate estimates that tend to underestimate the resistance of cells under more physiologically relevant conditions (87, 88). If our MICs are underestimates of resistance, then we would anticipate more false-positive evidence of competition in our experimental community. A second possibility for divergent results is the presence of physiological or evolutionary adaptation to the community conditions, across the 10 days of serial passaging. The stability and repeatability across replicates in Fig. 2 argue against a major role for genetic evolution in steering community dynamics—consistent with recent work on the suppressive impact of community interactions on bacterial evolution (89).

In order to develop an experimentally tractable model, we made a number of choices regarding specific experimental conditions (e.g., nutrients, initial community structure, strain identity) that likely influenced our specific results. The healthy lung is evidently an oxygen-rich environment; however, during the course of tissue degradation in the CF airways, the sputum environment can become oxygen deprived due to the combined forces of mucus plugs, along with oxygen consumption by immune cells and microbes (60, 61, 90). To capture an oxygen-stressed environment, we performed our experiments under static anaerobic conditions that were only subjected to oxygenation during bench passaging every 48 h. While all bacteria in the community are capable of either fermentation, anaerobic respiration, or both, the largely anaerobic condition represents a potential to bias the results toward strictly anaerobic bacteria. Our clinical benchmarking exercise indicates that the distribution of oxygen exposures in the clinic is less biased toward anaerobic conditions, as our three endpoint taxon misses (Fig. 10) consisted of overrepresentation of an anaerobe (*P. melaninogenica*) and underrepresentation of two aerobes (P. aeruginosa, *R. mucilaginosa*). This pattern is also consistent with recent transcriptomic analyses of P. aeruginosa from CF sputum, highlighting a transcriptional response indicative of reduced oxygen, but not necessarily anaerobic conditions (91). In future work we will investigate synthetic community dynamics in static communities with partial exposure to room air, following recent experimental *ex vivo* (patient sputum) models (92, 93).

Turning to our choices regarding synthetic community composition, by focusing on the most abundant bacterial taxa, we ignored the potential for rare keystone species to shape community dynamics (94). We also overlooked the potential importance of interactions among strains within each species (95, 96). Concerning specific strain choices, Fig. 3 illustrates that replacing mucoid P. aeruginosa PDO300 with an otherwise isogenic nonmucoid strain (PAO1) produces little dynamical change. However, other studies in different environmental contexts have demonstrated substantial dependency of interactions on strain identity (97, 98), leaving open the importance of specific strain identities in governing community outcomes. More broadly, we did not include other potentially critical players in the lung microbiome, spanning human epithelial and immune cells, fungal species, and viruses of all the above. We note that our experimental platform is amenable to the addition of these players and additional manipulation of timing and order of introductions in future controlled experiments.

Our results demonstrate the power of a model 10-species system for the study of chronic lung infection dynamics. This model provides a platform to assess the community ecological impacts of currently deployed antibiotic treatments (Fig. 4 and 8) and novel treatments—from different compounds to different strategies of their implementation. Current practice is to hit hard with an antibiotic that is effective against a target pathogen (99). In the context of our model community, detecting drug-susceptible P. aeruginosa would typically trigger combination treatments that lead in our example to rapid emergence of more dominant and more resistant pathogen replacements (Fig. 4 and 8) (100). One avenue to improve on this picture is to run community-scale resistance diagnostics and, in turn, use this diagnostic information to optimize antibiotic (and probiotic) choices (101). While simple in outline, identifying optimal treatment choices in the context of complex multispecies communities poses a substantial computational and experimental challenge.

## MATERIALS AND METHODS

### Bacterial strains.

Table 1 outlines the specific strains in our 10-species community. Species choices were initially informed based on our previous study of a 77-person CF cohort with samples taken during periods of clinical stability (57). Our 10 species represent the most abundant genera from our 16S rRNA analyses (together accounting for over 85% of reads). Note that these species are collectively representative of the “metacommunity” (the community of communities [102]) of microbes across a population of people with CF and are not necessarily representative of individual community states. We view this metacommunity as the menu of organisms from which individual communities are sampled.

**TABLE 1:** Experimental model organisms used in synthetic community experiments

Species	Exptl strain	Relative abundance of the genus in clinical samples (%)
**Pseudomonas aeruginosa**	PDO300 (mucoid) PAO1 (wild type)	29.7
Veillonella parvula	Clinical^*a*^	9.8
Rothia mucilaginosa	ATCC 49042^*b*^	9.1
Prevotella melaninogenica	ATCC 25845^*b*^	8.4
Streptococcus mitis	ATCC 49456^*c*^	7.9
**Haemophilus influenza** **e**	ATCC 10211	5.8
**Staphylococcus aureus**	SAJE2	5.6
**Achromobacter xylosoxidans**	ATCC 27061	4.8
Neisseria subflava	ATCC 49275^*c*^	1.8
**Burkholderia cenocepacia**	K56-2	1.1

a Isolate from Children’s Hospital of Atlanta. Collectively, these organisms represent over 85% of clinical sequence reads across a 77-person CF lung microbiome study (57).

b Pulmonary source.

c Oral source. Bold font indicates established CF pathogen (14).

To guide our experimental species choices, we turned to existing CF metagenome sequencing data (103), which provided high confidence for all but one of our species calls (Table 1). The exception is Streptococcus, where reads are distributed across a range of species. We chose Streptococcus mitis because it is present in sputum metagenomic profiles (103), and it is an experimentally tractable organism that is typically considered to be nonpathogenic (104). Within each species, we focused on well-characterized reference strains, as far as these were available, including American Type Culture Collection (ATCC) strains. For the dominant pathogen, P. aeruginosa (PA), we used both the reference strain PAO1 and its mucoid derivative PDO300 (105). Our default experimental choice is PDO300, as this strain better reflects the mucoid phenotype prevalent in chronic CF (105, 106).

### Community growth medium.

Our artificial sputum medium (ASM) is based on the benchmarked synthetic CF sputum medium 2 (SCFM2 [55, 56]), but with differences in the preparation of the mucin and DNA macromolecules. Specifically, mucins were ethanol washed and autoclaved (not UV sterilized, due to larger volume requirements), and the entire medium was filter sterilized following addition of DNA. Given the potential for differences in preparation methods to impact the results, we refer to our medium under the more generic name of ASM to underline these differences from the reference recipe for SCFM2 (55, 56).

### Bacterial preculture and community construction.

Before the experiment, all bacterial strains were revived from frozen stocks by streaking on rich medium agar plates (chocolate or brain heart infusion [BHI] agar, depending on the species; see Table S4) and cultured at 37°C for 48 h microaerophilically (for H. influenzae and *N. subflava*) or anaerobically (for *P. melaninogenica* and *V. parvula*, in GasPak jars). Five colonies were then picked from each plate and used to inoculate specific monoculture rich medium, which was cultured for a further 48 h; specific culture conditions are detailed in Table S4.

10.1128/msphere.00318-22.4 TABLE S4 Monoculture preculture conditions. The atmospheric environment specifies the oxygenation used for both the agar plate (brain heart infusion [BHI] or chocolate agar) and liquid culture steps. The liquid medium supplements had the following concentrations: hemin, 15 mg/L; NAD, 15 mg/L; vitamin K1, 1 mg/L; l-lactate, 50 mM. Note that in subsequent experiments we simplified the protocol so that all bacteria were cultured first on chocolate agar plates and then in a common medium of TSYE supplemented with hemin, NAD, vitamin K, and lactic acid. Download Table S4, DOCX file, 0.01 MB. Copyright © 2022 Varga et al. 2022 Varga et al. https://creativecommons.org/licenses/by/4.0/ This content is distributed under the terms of the Creative Commons Attribution 4.0 International license.

The bacterial cultures were then washed in a defined ASM buffer base (ASM minus all carbon sources), and optical density at 600 nm (OD_600_) values were measured with a Hidex plate reader (Hidex Oy, Finland) and adjusted to 0.5 for each species and diluted 10-fold in ASM. These standardized bacterial dilutions of equal volume were mixed, and antibiotic stocks were added according to the experimental design for each treatment. The bacterial mixtures (plus antibiotics, dependent on treatment) were homogenized with pipetting and then divided into five replicates of 2 mL each in 24-well plates. An additional 0.5 mL of the initial inoculum mixture was stored at −80°C to assess community composition at time 0 by subsequent genomic analysis.

### Treatments and passaging.

To measure the impact of exposure to antibiotics, we tested three antibiotics that are widely used in CF therapy, tobramycin (5 μg/mL), meropenem (15 μg/mL), and ciprofloxacin (2.5 μg/mL), and two widely used combinations, tobramycin and meropenem and tobramycin and ciprofloxacin (adding the concentrations above). The specific concentrations used reflect measurements of antibiotic concentrations in CF sputum (68–72). Our choice of 5 μg/mL tobramycin is low compared to peak concentrations measured immediately following inhaled therapy (107, 108). Even in this immediate posttreatment context, the concentrations we used are within the range of their reported measured concentrations at 30 min posttreatment (108). Concentrations are not reported for any longer duration in these studies. All experiments were performed with 5 replicates of 2-mL cultures in 24-well plates cultured at 37°C in anaerobic GasPak jars. Every 48 h, bacterial cultures were mixed by pipetting, and 10% of the volume was transferred to fresh ASM (with fresh antibiotics as defined by the treatment). Then, 0.5 mL of the culture was stored at each passage at −80°C for later DNA purification and amplicon sequencing. Each experimental line was maintained for 5 passages (10 days).

To assess the role of pathogen characteristics, we conducted 5 pathogen manipulations (presence/absence of P. aeruginosa mucoidy [PDO300 versus PAO1] × presence/absence of S. aureus, plus a no P. aeruginosa *+* no S. aureus treatment). These experiments were done in the absence of antibiotics but otherwise with the same conditions as described above.

### 16S rRNA sequencing and qPCR.

DNA purification, sequencing, and quantitative PCR (qPCR) were performed by MR DNA Lab (Shallowater, TX). Briefly, DNA was purified from sputum homogenate after mechanical lysis with the power soil kit (MoBio, Carlsbad, CA). The 16S V4 region of the resulting DNA was amplified with 515F and 806R primers incorporating the barcode in the forward primer and subjected to Illumina sequencing (109). The sequence data were generated in a total of 6 MiSeq runs. Total 16S abundance in each sample was determined by qPCR using standard 515F/806R primers (109).

### 16S rRNA sequence analysis.

To generate taxa counts from the sequence data, we processed each run independently and combined the results. Across the 6 sequencing runs, a total of 15,347,658 sequence reads were generated, with a median of 59,686 sequences per sample (minimum 22,707, maximum 126,680). All sequence processing was done through QIIME2 2019.10.0. Unless otherwise noted, we left parameters as defaults based on the Moving Pictures workflow. Samples were demultiplexed using the cutadapt plugin in QIIME2. We found that some of the barcode sequences were also found in the 16S region of several taxa. To mitigate this confounder, we removed from each metadata file the first four nucleotides in the 515F primer and added them to the barcode. For example, the barcode GAGATGTG was remapped as GAGATGTGGTGC and the primer became CAGCMG….

Reads were denoised using the deblur plugin, and the resulting sequences were trimmed to 250 bp. Taxonomic assignments were classified against the greengenes 16S database. Some assignments were not possible at a level of genus resolution, so we interpreted reads mapping to “o__Lactobacillales” to “g__Streptococcus,” “f__Burkholderiaceae” as “g__Burkholderia,” and “f__Pseudomonadaceae” as “g_Pseudomonas.” Finally, for each sample we removed spurious (and rare) taxon calls that did not map onto our experimentally defined communities.

Absolute abundances were determined by the proportion of the total 16S count and then normalized to species-specific 16S rRNA copy counts (57, 110).

### Data availability.

Sequence data have been deposited to the SRA (accession project number PRJNA752117). The analysis pipeline is available on GitHub (github.com/GaTechBrownLab/Varga-et-al_CompetitiveAbxRelease_SRA-upload).

### Statistical analyses.

All analyses and plots used the R programming language (111, 112). Tables and scripts can be found at (https://github.com/GaTechBrownLab/Varga-et-al_CompetitiveAbxRelease_SRA-upload). A nonparametric Wilcoxon rank sum test was used to test for differences in absolute species abundances across experimental conditions, using a two-tailed test for change in abundance and a one-tailed test to assess competitive release (testing for increases only). A *t* test with a Bonferroni multiple testing correction was performed to compare relative abundances of the 10 species under experimental conditions with clinical samples from the 77-patient cohort. To compare experimental treatments (and clinical benchmark data) at a community scale, we calculated ANOSIM *R* values on Bray-Curtis dissimilarity matrices for each treatment using the vegan package (113–115). The R statistic is a ratio of within-treatment differences to between-treatment differences on a scale of −1 to 1, where a value of 1 would mean that all dissimilarity is between treatments, indicating completely different communities.

To visualize community-scale differences, we constructed ordination plots for combined clinical and experimental compositional data. Clinical and experimental observations were center-log-transformed first (116, 117) and then standardized before principal-component analysis (111, 117).
