# Supplementary material for: Antibiotics Drive Expansion of Rare Pathogens in a Chronic Infection Microbiome Model
Source: mSphere. 2022 Aug 16;7(5):e00318-22. doi: 10.1128/msphere.00318-22 (PMC9599657; doi:10.1128/msphere.00318-22)
Supplement: TABLE S1 [file msphere.00318-22-s0002.docx]

**Table. S1. Differences in community structures across pathogen treatments (Figure 3 data).** The analysis of similarity (ANOSIM R) statistic captures the ratio of between-group to within-group variances; as R approaches 1, more variance is found between groups than within groups^112^. Passage 0 (inoculum) is omitted from the analyses. All but one R value is significant via permutation tests at *p* < 0.05. Applying the more conservative convention of *R >* 0.4 for significance^118,119^, we find no significant effects of any pathogen treatment.

| **Treatment contrast** | **ANOSIM *R* (permutation *p*value)** |
| --- | --- |
| Effect of mucoidy (SA present) | *R =*0.135 (*p =*0.003) |
| Effect of mucoidy (SA absent) | *R =*0.019 (*p =*0.164) |
| Effect of SA removal (mucoid PA) | *R =*0.068 (*p =*0.026) |
| Effect of SA removal (non-mucoid PA) | *R =*0.080 (*p =*0.010) |
| Effect of SA and PA removal (mucoid PA) | *R =*0.193 (p = 0.001) |
