# Supplementary material for: Antibiotics Drive Expansion of Rare Pathogens in a Chronic Infection Microbiome Model
Source: mSphere. 2022 Aug 16;7(5):e00318-22. doi: 10.1128/msphere.00318-22 (PMC9599657; doi:10.1128/msphere.00318-22)
Supplement: TABLE S2 [file msphere.00318-22-s0007.docx]

Table S2. Antibiotic susceptibility in rich medium. Minimal Inhibitory Concentrations (MICs, in μg / ml) of synthetic community members were determined in rich medium.

|  | Tobramycin | Meropenem | Ciprofloxacin |
| --- | --- | --- | --- |
| *P. aeruginosa* PAO1 | 1 | 1 | 0.125 |
| *P. aeruginosa* PDO300 | 2 | 1 | <0.125 |
| *S. aureus* | 8 | 1 | 64 |
| *B. cenocepacia* | $\geq$ 128 | 32 | 32 |
| *A. xylosoxidans* | $\geq$ 128 | 4 | 8 |
| *S. mitis* | 4 | 0.25 | 2 |
| *N. subflava* | 8 | 0.125 | 0.125 |
| *R. mucilaginosa* | 128 | 0.125 | 16 |
| *H. influenzae* | 4 | 0.125 | 0.125 |
| *P. melaninogenica* | $\geq$ 128 | 0.125 | 1 |
| *V. parvula* | 32 | 0.25 | 1 |
