# Supplementary material for: Antibiotics Drive Expansion of Rare Pathogens in a Chronic Infection Microbiome Model
Source: mSphere. 2022 Aug 16;7(5):e00318-22. doi: 10.1128/msphere.00318-22 (PMC9599657; doi:10.1128/msphere.00318-22)
Supplement: TABLE S3 [file msphere.00318-22-s0008.docx]

**Table S3. Summary of hypothesis tests conducted in this study.** Hypotheses (italic text) are organized by topic area (underlined text). Hypotheses in grey are rejected under the specific experimental conditions outlined in our study, and may still apply in other contexts. Previous literature citations identifies work that is broadly relevant to the topic identified, and citations do not imply support for the specific hypotheses outlined.

| **Topic and *hypotheses* (grey/black – rejected/supported under our specific experimental conditions)** | **Previous literature** | **Key figures** |
| --- | --- | --- |
| Synthetic microbiome structure and diversity  *Single-locus biofilm mutations can produce large-scale community shifts.* | *^66^* | 3 |
| Competitive release / survival filter  *Antibiotics enrich for resistant species (taxon enrichment).*  *Antibiotics enrich for the sum of resistant species (functional enrichment).* | ^75-77^ | 4, 6, 8 |
| Variation and alternative stable states  *Drug exposure increases variability across replicates*  *Drug exposure produces alternate stable states* | ^74^ | S1, S3 |
| Role of oral bacteria in CF microbiomes  *Oral bacteria facilitate CF pathogens.*  *Oral bacteria suppress CF pathogens.* | ^20,26^ | 2, 4 |
| Microbial interactions  *Stressors increase inter-specific facilitation.*  *B cenocepacia* facilitates *S. aureus* in a meropenem-dependent manner. | ^122^ | 7 |
| Models of CF microbiomes  *Distinct experimental platforms are necessary to produce distinct ‘pulmotypes’.*  *A single ‘meta-community’ experimental platform can approach diverse CF ‘pulmotypes’, contingent on antibiotic exposures.* | ^54^ | 4, 5, 9, 10 |

**Reference**

122. Piccardi P, Vessman B, Mitri S. 2019. Toxicity drives facilitation between 4 bacterial species. Proc Natl Acad Sci U S A 116:15979–15984. <https://doi.org/10.1073/pnas.1906172116>.
