# Supplementary material for: Antibiotics Drive Expansion of Rare Pathogens in a Chronic Infection Microbiome Model
Source: mSphere. 2022 Aug 16;7(5):e00318-22. doi: 10.1128/msphere.00318-22 (PMC9599657; doi:10.1128/msphere.00318-22)
Supplement: TABLE S4 [file msphere.00318-22-s0009.docx]

**Table S4. Monoculture pre-culture conditions.** The atmospheric environment specifies the oxygenation used for both the agar plate (Brain Heart Infusion (BHI) or chocolate agar) and liquid culture steps. The liquid medium supplements had the following concentrations: hemin, 15 mg / L; NAD, 15 mg / L; vitamin K1, 1 mg / L; L-lactate, 50 mM. Note that in subsequent experiments we simplified the protocol so that all bacteria were cultured first on chocolate agar plates, and then in a common medium of TSYE supplemented with hemin, NAD, vitamin K, and lactic acid

| Genus | Species | Oxygen tolerance | Liquid medium | Agar plates |
| --- | --- | --- | --- | --- |
| *Pseudomonas* | *aeruginosa* | Aerobic | TSYE | BHI |
| *Staphylococcus* | *aureus* | Aerobic | TSYE | BHI |
| *Achromobacter* | *xylosoxidans* | Aerobic | TSYE | BHI |
| *Haemophilus* | *influenzae* | Microaerophilic | TSYE+hemin+NAD | Chocolate agar |
| *Streptococcus* | *mitis* | Aerobic | TSYE | BHI |
| *Rothia* | *mucilaginosa* | Aerobic | TSYE | Chocolate agar |
| *Burkholderia* | *cenocepacia* | Aerobic | TSYE | BHI |
| *Neisseria* | *subflava* | Microaerophilic | TSYE | Chocolate agar |
| *Prevotella* | *melaninogenica* | Anaerobic | TSYE+hemin+VK1 | Chocolate agar |
| *Veillonella* | *parvula* | Anaerobic | TSYE+lactate | Chocolate agar |
